# CCL20/TNF/VEGFA Cytokine Secretory Phenotype of Tumor-Associated Macrophages Is a Negative Prognostic Factor in Cutaneous Melanoma

**DOI:** 10.3390/cancers13163943

**Published:** 2021-08-05

**Authors:** Alba Gutiérrez-Seijo, Elena García-Martínez, Celia Barrio-Alonso, Miriam Pareja-Malagón, Alejandra Acosta-Ocampo, María Eugenia Fernández-Santos, Amaya Puig-Kröger, Verónica Parra-Blanco, Enrique Mercader, Iván Márquez-Rodas, José Antonio Avilés-Izquierdo, Rafael Samaniego, Paloma Sánchez-Mateos

**Affiliations:** 1 Unidad de Microscopía Confocal, Instituto de Investigación Sanitaria Gregorio Marañón, 28007 Madrid, Spain; albaguti@ucm.es (A.G.-S.); egarciamartinez2@salud.madrid.org (E.G.-M.); celibarr@ucm.es (C.B.-A.); 2 Laboratorio de Inmuno-Oncología, Instituto de Investigación Sanitaria Gregorio Marañón, 28007 Madrid, Spain; 3 Unidad de Producción Celular, Instituto Investigación Sanitaria Gregorio Marañón, 28007 Madrid, Spain; miriam.pareja@iisgm.com (M.P.-M.); alejandra.acosta@iisgm.com (A.A.-O.); mariuge@fibhgm.org (M.E.F.-S.); 4 Laboratorio de Inmuno-Metabolismo e Inflamación, Instituto de Investigación Sanitaria Gregorio Marañón, 28007 Madrid, Spain; amaya.puig@iisgm.com; 5 Servicio de Anatomía Patológica, Hospital General Universitario Gregorio Marañón, 28007 Madrid, Spain; veronica.parra@salud.madrid.org; 6 Unidad Cirugía Endocrino-Metabólica, Servicio de Cirugía General y Aparato Digestivo, Hospital General Universitario Gregorio Marañón, Calle Doctor Esquerdo 46, 28007 Madrid, Spain; enrique.mercader@salud.madrid.org; 7 Laboratorio Inmunofisiología, Instituto de Investigación Sanitaria Gregorio Marañón, Calle Doctor Esquerdo 46, 28007 Madrid, Spain; 8 Servicio de Oncología Médica, Hospital General Universitario Gregorio Marañón, 28007 Madrid, Spain; ivan.marquez@salud.madrid.org; 9 Servicio de Dermatología, Hospital General Universitario Gregorio Marañón, 28007 Madrid, Spain; jantonio.aviles@salud.madrid.org; 10 Departamento de Inmunología, Facultad de Medicina, Universidad Complutense de Madrid, 28040 Madrid, Spain

**Keywords:** CCL20, TNF, VEGFA, melanoma, metastasis, TAM, biomarker, prognostic factor

## Abstract

**Simple Summary:**

Cutaneous melanoma is characterized by its heterogeneous metastatic behavior and robust biomarkers are still needed to identify those patients with increased risk for distant metastasis, to guide new adjuvant treatments. We aimed to assess the prognostic role of different features of tumor-associated macrophages (TAMs) using multicolor immunofluorescence microscopy and single-cell analysis. Rather than the number, size, or location of TAM, quantitative assessment of CCL20, TNF, and VEGFA cytokine content was associated with strong prognostic significance in primary melanoma. This novel TAM cytokine signature serves as a readout of TAM prometastatic activation and provides independent information to traditional TNM melanoma staging. In addition, we show that this particular cytokine profile is coregulated by p53 and NF-κB, suggesting that therapies targeting both pathways may modulate the prometastatic deviation of TAMs in melanoma.

**Abstract:**

TAMs constitute a large fraction of infiltrating immune cells in melanoma tissues, but their significance for clinical outcomes remains unclear. We explored diverse TAM parameters in clinically relevant primary cutaneous melanoma samples, including density, location, size, and polarization marker expression; in addition, because cytokine production is a hallmark of macrophages function, we measured CCL20, TNF, and VEGFA intracellular cytokines by single-cell multiparametric confocal microscopy. The Kaplan–Meier method was used to analyze correlation with melanoma-specific disease-free survival and overall survival. No significant correlations with clinical parameters were observed for TAM density, morphology, or location. Significantly, higher contents of the intracellular cytokines CCL20, TNF, and VEGFA were quantified in TAMs infiltrating metastasizing compared to non-metastasizing skin primary melanomas (*p* < 0.001). To mechanistically explore cytokine up-regulation, we performed in vitro studies with melanoma-conditioned macrophages, using RNA-seq to explore involved pathways and specific inhibitors. We show that p53 and NF-κB coregulate CCL20, TNF, and VEGFA in melanoma-conditioned macrophages. These results delineate a clinically relevant pro-oncogenic cytokine profile of TAMs with prognostic significance in primary melanomas and point to the combined therapeutic targeting of NF-kB/p53 pathways to control the deviation of TAMs in melanoma.

## 1. Introduction

Despite the significant survival benefit provided by new treatments, metastatic melanoma continues to be a life-threatening disease [1]. At the time of clinical presentation, skin melanomas are frequently excised when distant metastases are clinically undetectable and most patients are included in stage I and II (localized melanomas) or stage III (regional disease) [2]. A small proportion of cases (<4%) show distant metastases (stage IV) at the time of diagnosis of the primary tumor, whereas most metastatic cases progress from stages II-III patients during follow-up. Identification of patients with biologically aggressive melanomas is important because treatment at an earlier clinical stage improves disease-free survival but with the risk of toxicity [3]. With survival rates ranging from 63–81% in stage II and 36–63% in stage III [4], there is great variability in metastatic risk at diagnosis and robust biomarkers are currently needed. Some tumor microenvironment (TME)-derived factors or infiltrating cells, particularly immune cells, are a source of biomarkers, providing independent prognostic information for assessing metastatic risk or to predict the efficacy of new treatments [5]. 

Tumor-associated macrophages (TAMs) are among the most frequent immune infiltrating cells in solid tumors, displaying a variety of phenotypes and functions [6]. Single-cell analysis in lung adenocarcinoma and renal cancer has revealed an unsuspected heterogeneity of TAM phenotypes [7,8]. Diversity of macrophages was oversimplified in the M1/M2 classification, in which skewing TAMs towards M1-type may result in anti-tumor responses whereas M2 phenotype promotes tumor evolution and metastasis [9]. Characterization of macrophage polarization in human tissues is an important issue; however, due to their complexity and to differences with studies mostly performed in vitro, it has remained difficult [10]. The use of single markers as currently performed by most immunohistochemistry approaches (IHC) also prevents correct identification of M1/M2 cell subsets or differentiation from other mononuclear phagocyte populations [11]. In advanced solid tumors, TAMs are known to be preferentially M2 biased; however, cutaneous melanomas are generally non-advanced primary tumors at diagnosis and M1/M2 subsets remain to be properly addressed in large patient groups [12]. Several studies have evaluated TAM number/density as prognostic factors for stage I/II melanomas with opposite results. Jensen et al. [13], using the general marker of human macrophages CD68 and CD163 as a single M2 marker, showed that macrophage infiltration at the invasive front was an independent predictor of poor survival. Subsequently, other large-scale studies did not corroborate the correlation between CD68 positive TAM number and melanoma survival [14,15].

Complex paracrine interactions among tumor cells and a variety of non-tumoral components and infiltrating cells take place in the TME and impact tumor biology [16]. We previously reported that in human melanoma the chemokine axis CCR6/CCL20 is involved in a cooperative paracrine loop between CCR6 expressing tumor cells and CCL20 secreted by nontumoral cells in the stroma [17]. Importantly, we showed that stromal CCL20 predicted poor survival in a cohort of 40 primary melanoma patients and identified TAMs as the main stromal source of CCL20 in melanoma tissues. Thus, we hypothesize that the CCL20 secretory TAM phenotype might be associated with melanoma progression and could be used for an accurate prognosis of metastatic risk in skin melanoma. In the current study, we measured diverse characteristics of tissue TAMs, including CCL20 and other cytokines by multicolor immunofluorescence and single-cell analysis, and correlated them with the clinical outcome of cutaneous melanoma patients.

## 2. Materials and Methods

### 2.1. Study Cohorts and Selection Criteria

All clinical data and patient samples were collected following approval by the Hospital Gregorio Marañón ethics committee and informed consent was obtained for each patient. A frozen-preserved collection of skin melanomas (punch biopsies not compromising patient diagnosis) and corresponding patient data were previously described [17] (Table S1). We also retrieved 83 formalin-fixed and paraffin-embedded (FFPE) primary cutaneous melanomas; with a median patient follow-up of 77 ± 47 months, >2 mm Breslow thickness, excised between 1998 and 2015 in our institution; with either clinically aggressive disease developing distant metastasis or lymph node recurrence during follow-up (51 metastasizing primary melanomas, with 33/51 melanoma-related and 2/51 non-related deaths) or matched skin melanoma samples from patients who were disease-free for at least 10 years of follow-up (32 non-metastasizing primary melanomas). Pathological AJCC staging II-IV assessment was obtained by sentinel lymph node biopsy and distant metastasis evaluation by computed tomography at the time of diagnosis. Follow-up studies were performed every 6 months for 5 years and then annually up to 10 years unless there was an earlier documented metastatic event (regional or distant metastasis). Patients were selected because they were representative of opposite clinical outcomes during follow-up, therefore, primary tumors were thicker (median and mean, 3.9 mm and 4.9 mm, respectively) than in a population-based registry data [18]. Metastasizing and non-metastasizing primary tumors had comparable Breslow thickness (mean, 5.3 mm and 4.2 mm, respectively; Mann–Whitney, *p* = 0.47). Six patients with immediate recurrence after the first diagnosis were excluded for disease-free survival (DFS) but not for overall survival (OS) (Table S1).

### 2.2. Multicolor Fluorescence Confocal Microscopy

FFPE sections were de-paraffinized, rehydrated, and unmasked by steaming in 10 mM sodium citrate buffer pH 6.0 (Dako) for 6 min. Slides were blocked with 5 μg/mL human immunoglobulins (Ig) solved in blocking serum-free medium (Dako) for 30 min, and then sequentially incubated with 5–10 μg/mL primary antibodies specific for CCL20 (Abcam ab9829, polyclonal rabbit IgG), TNFα (Abcam ab1793, clone 52B83, mouse IgG1), VEGFA (Antibodies online, ABIN191867, rabbit IgG), CD115 (R&D AF329, polyclonal Goat IgG), CD163 (Bio-Rad MCA1853T, clone EDHu-1, mouse IgG1) and/or CD68 (Dako, clone PG-M1, mouse IgG3) in phosphate buffer solution (PBS) containing 10% blocking medium overnight at 4 °C, and then proper fluorescent secondary antibodies (Jackson-Immunoresearch, Invitrogen) for 1 h at room temperature. Intermediate washes were performed in agitation and by immersion in PBS containing 0.05% Tween-20. Samples mounted with fluorescent mounting medium (Dako) were imaged with a Leica SPE confocal microscope using a glycerol-immersion ACS APO 20×/NA 0.60 objective. Cytokines used in immunocompetition assays were purchased from Immunotools.

Single-cell quantification was performed in 3–5 20× fields, as previously described [17,19,20]. For proper TAM segmentation, whole-cell area staining is necessary to define macrophages contour, which is best achieved by CD163 staining in frozen samples fixed with acetone, and CD68 labeling in formalin-fixed paraffin-embedded sections. Mean fluorescence intensity (MFI) of proteins of interest was obtained at manually depicted TC nests or at each segmented TAM using the ‘analyze particle’ plugging of FIJI software. For TAM density, 3–5 fields were quantified, discriminating two different regions. Intratumoral regions corresponded to identifiable tumor nests, whereas stromal regions were characterized by elongated DAPI stained nuclei, including stromal bundles wider than 30 µm and peritumoral areas up to 300 µm far from melanoma cells. Only regions clearly differentiable were included in the study.

### 2.3. Cell Isolation from Human Melanomas 

Biopsies from stage IV melanoma patients were homogenized and digested into single-cell suspensions (Tumor Dissociation Kit, Miltenyi, Bergisch Gladbach, Germany). TAMs and tumor-infiltrating lymphocytes were purified by magnetic cell sorting using CD14 and CD3 microbeads, respectively. The remaining negatively selected cells were considered as ‘TCs’ [17,19]. 

### 2.4. Monocyte Isolation and Cell Culture

Peripheral blood mononuclear cells (PBMCs) were isolated from patients or buffy-coats from healthy donors over a ficoll gradient (Lymphocytes Isolation Solution, Rafer). Monocytes were purified by magnetic cell sorting using anti-CD14 tagged microbeads (Miltenyi Biotech, Bergisch Gladbach, Germany). For in vitro generation of macrophages, monocytes were cultured at 0.5 × 10^6^/mL for 7 days containing GM-CSF (10 ng/mL or 1,000 U/mL, Immunotools) or M-CSF (10 ng/mL, Immunotools) to generate MP-GM or MP-M macrophages, respectively. Cytokines were added every two days. All cells, including melanoma cell lines BLM and A375 [21] were cultured in RMPI-1640 medium (Gibco, Waltham, MA, USA) supplemented with 10% fetal calf serum (FCS, Sigma, Burlington, MA, USA). 

### 2.5. In Vitro Measurements

Melanoma cells were cocultured with macrophages at 1:2 ratios (melanoma/macrophage). After 24 and 72 h, supernatants were collected for secreted proteins assessment (CCL20, TNF, and VEGFA ELISAs, R&D Systems), and magnetically separated cells were processed for quantitative real-time PCR (qPCR) analyses. When specified, cultures contained 10 µM BAY11-7082 (NF-κB-inhibitor, ChemCruz) and/or 50 µM Pifithrin-α (p53-inhibitor, Sigma).

For qPCRs, oligonucleotides (CCL20, S 5′ gctgctttgatgtcagtgct; AS 5′ gcagtcaaagttgcttgctg; TNF, S 5′ cagcctcttctccttcctgat; AS 5′ gccagagggctgattagaga; VEGFA, S: 5′ gcagcttgagttaaacgaacg; AS: 5′ ggttcccgaaaccctgag) were designed according to the Roche software for qPCR. Total RNA was extracted (NucleoSpin RNA-purification kit, Macherey Nagel, Düren, Germany) and retrotranscribed cDNA quantified using the Universal Human Probe Roche library (Roche-Diagnosis, Basel, Switzerland). Assays were made in triplicate and normalized to TBP and/or HPRT1 expression (∆∆CT method). For RNAseq and Gene Set Enrichment Analysis (GSEA), total RNA was isolated from three independent preparations and processed at BGI (https://www.bgi.com, accessed on 4 August 2021), where library preparation, fragmentation, and sequencing were performed using the DNBseq platform. An average of 4.39 Gb bases were generated per sample and, after filtering, clean reads were mapped to the reference (UCSC Genome assembly hg38) using Bowtie2 (average mapping ratio 95.49%) [22]. Gene expression levels were calculated by using the RSEM software package [23]. Differential gene expression was assessed by using DEseq2 algorithms using the parameters Fold change >2 and adjusted *p*-value < 0.05. For gene set enrichment analysis (GSEA) (http://www.broad.mit.edu/gsea/, accessed on 4 August 2021), the gene sets available at the website [24], as well as the “HALLMARK_P53_PATHWAY” and “HALLMARK_TNFA_SIGNALING_VIA_NFKB” gene sets, that contain the top genes involved in p53 and NF-kB pathways respectively, were used [25]. The data discussed in this publication have been deposited in NCBI’s Gene Expression Omnibus [26] and are accessible through GEO Series accession number GSE171277.

### 2.6. Statistical Analyses 

Censured Kaplan-Meier curves were used to analyze the correlation with patient disease-free and overall survival, and the Cox-regression method (univariate and multivariate) to identify independent prognostic variables. Mann-Whitney t-test was used to evaluate the association with clinicopathological features. The paired t-test, Spearman R correlation, and Log-rank analyses have also been used in this study (Graph-Pad software, San Diego, CA, USA), as indicated. *p* < 0.05 was considered statistically significant.

## 3. Results

Motivated by the idea of identifying prognostic biomarkers derived from the TME, we sought to identify and quantify diverse TAM subsets/characteristics in two independent collections of primary melanoma samples with clinical information and the survival status of the patients. Table S1 lists the clinicopathological characteristics of 40 cryopreserved primary melanomas collected in a prospective patient cohort, and 83 FFPE samples retrieved from the archive, as described in Material and Methods. Primary melanomas were classified as non-metastasizing or metastasizing regarding subsequent development of metastasis during patient follow-up of 5 and 10 years in each cohort, respectively. 

**Figure 1:**
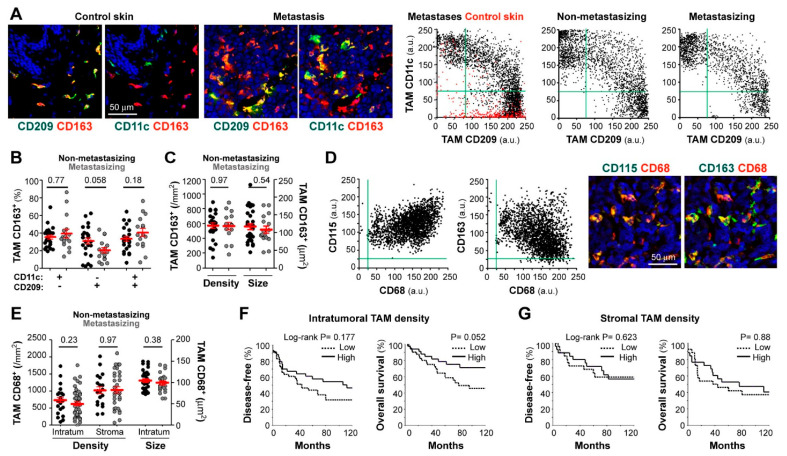
Correlation between patient evolution and TAM density, size, and polarization state. Confocal microscopy screening of two independent and differently preserved primary melanoma collections: frozen (stage I-III; (**A**–**C**)) and FFPE (stage II-IV; **D**–**G**). (**A**) Frozen tissues co-stained for CD163 (red) and CD11c or CD209 (green), as indicated. Dot-plots show single-cell MFI (arbitrary units, a.u.) for CD11c and CD209 proteins simultaneously quantified at CD163^+^ TAMs in control skin (*n* = 3, red dots), skin metastases (*n* = 6), non-metastasizing (*n* = 24) and metastasizing (*n* = 11) primary melanomas. (**B**) Relative percentages of polarized TAMs through the whole cryopreserved primary collection (*n* = 40). (**C**) Average density (by mm^2^) and size (µm^2^) of CD163^+^ TAMs quantified through intratumoral and/or peritumoral regions of primary melanomas (*n* = 40). (**D**) Paraffin-embedded melanoma co-stained for pan-macrophage markers CD68 (red) and CD163 or CD115 (green), as indicated. Dot-plots show single-cell expression of CD68 vs. CD115 or CD163 proteins quantified in either CD68^+^, CD163^+^ and/or CD115^+^ TAMs (*n* = 3 non-metastasizing and 3 metastasizing primary melanomas). (**E**) CD68^+^ TAM density quantified through intratumoral (*n* = 83) and stromal (*n* = 54) areas of primary melanomas. TAM size at intratumoral regions (*n* = 83), are shown. (**F**,**G**) Correlation between patient evolution and CD68^+^ TAM density at intratumoral (**F**) and stromal (**G**) regions. Disease-free and overall survival 10-year Kaplan–Meier curves are shown. Cut-off values were calculated from ROC curves to classify as ‘low’ or ‘high’ density. P values are shown (Log-rank). Mann-Whitney statistical analysis was used to compare non-metastasizing vs. metastasizing melanomas in (**B**,**C**,**E**) panels. Scale bars, 50 µm.

We first analyzed macrophage subsets in cryopreserved samples, using triple-color labeling with CD11c, CD209, and CD163, to quantify cells with macrophage morphology in normal skin, skin melanoma metastases, and primary melanoma tumors (non-metastasizing and metastasizing) (Figure 1A) [17]. CD163, well expressed by most tissue macrophages [11], was used to gate macrophages and to measure CD11c (M1 marker) and CD209 (M2 marker) expression levels in gated CD163^+^ cells (Figure 1A). Accordingly, subsets were defined as follows: a CD209^+^CD11c^−^ M2 subset, which was the only group detected in normal skin (depicted in red in control skin dot-plot), CD209^−^CD11c^+^ M1 subset, which appeared as a new population in both metastases and primary melanomas, and CD209^+^CD11c^+^ (mixed population) (Figure 1A,B). Comparative analysis of 24 non-metastasizing versus 16 metastasizing primary tumors showed no difference in CD209^−^CD11c^+^ (M1-like) and CD209^+^CD11c^+^ (M1/M2 mixed) subsets, with a tendency to less CD209^+^CD11c^−^ (M2-like) macrophages in metastasizing primary melanomas (*p* = 0.058) (Figure 1B), consistent with our previous pilot study with 5 non-metastasizing versus 5 metastasizing cases [17]. We next quantified TAM density, which is a common parameter used to test the clinical relevance of immune cell populations, and TAM size, which was reported to correlate with functional diversity and polarity and may provide prognostic significance [27]. However, we did not find significant differences in TAM density or size between non-metastasizing and metastasizing primary tumors (Figure 1C).

Because harvesting primary melanoma tissues for cryopreservation is only feasible when sufficient tumor is available and requires close coordination between clinicians, we set up conditions for multicolor staining of macrophage markers in routine FFPE diagnostic material. Triple staining with CD68, CD115 (macrophage growth factor receptor, CSF1R), and CD163 demonstrated co-expression by most cells with macrophage morphology (Figure 1D). There was negative correlation between CD68 and CD163 mean fluorescence intensity (MFI) (R= −0.36, *n* = 6, *p* < 0.001); however, neither CD163 nor CD68 nor CD115 defined separated M1/M2 macrophage subsets, indicating that they are pan-macrophage markers in melanoma. For image quantification, we chose CD68 for macrophage segmentation because very few CD68^+^ cells were negative for CD115 or CD163, indicating that very few cells other than macrophages were CD68^+^ in melanoma tissues. We quantified TAM density in full-face FFPE primary melanoma sections, finding no significant differences between 32 non-metastasizing and 51 metastasizing primary tumors, regarding stromal or intratumoral TAM density (Figure 1E). Furthermore, the size of intratumoral TAMs was not different between the two clinically divergent groups (Figure 1E). We next defined low and high TAM density (in tumor nests and stromal areas independently) using the best cut-off value extrapolated from the ROC curve (receiver operating characteristic) and calculated 10-years DFS and OS Kaplan-Meier survival curves (Figure 1F,G, for intratumoral and stromal TAMs, respectively), finding a tendency of high intratumoral TAM density towards a better patient survival (Figure 1F, *p* = 0.052).

**Figure 2:**
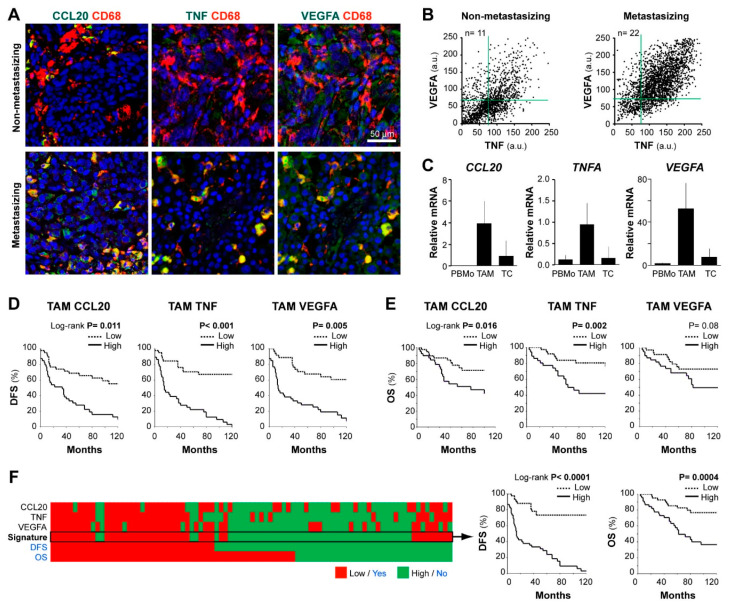
CCL20, TNF, and VEGFA expression in human melanoma TAMs and correlation with patient survival. (**A**) FFPE human melanoma samples co-stained for CD68 (TAM marker, red) and CCL20, TNF, or VEGFA (protumoral markers, green), as indicated. Representative images of non-metastasizing and metastasizing primary melanomas are shown. Scale bar, 50 µm. (**B**) Dot-plots showing CD68+TAMs from non-metastasizing (*n* = 11 samples; 1100 TAMs) and metastasizing (*n* = 22 samples; 2200 TAMs) primary melanomas. (**C**) Expression of *CCL20*, *TNFA*, and *VEGFA* mRNA (relative to average *TBP/HPRT1*) in freshly isolated peripheral blood monocytes CD14^+^ (PBMo), CD14^+^ TAMs, and negatively selected cells (CD14^−^CD3^−^) from three-stage IV primary melanoma patients. (**D**,**E**) Correlation between patient evolution and TAM expression of CCL20, TNF, and VEGFA proteins. Disease-free (**D**) and overall (**E**) survival 10-year Kaplan–Meier curves are shown. Median values of the 83 primary melanomas were used to classify as ‘low’ or ‘high’ expressing samples. *p* values are shown (Log-rank). (**F**) Kaplan–Meier curves using CCL20/TNF/VEGFA as a whole signature (classified as ‘high’ or ‘low’ by majority: ≥2/3). DFS, disease-free survival. OS, overall survival.

We previously identified co-expression of CCL20, TNF, and VEGFA cytokines by tissue TAMs in an exploratory set of 7 metastasizing melanomas [17]. Now, we tested the hypothesis that this distinctive prometastatic TAM secretory phenotype can be identified in routine FFPE samples, to be clinically applicable for patient profiling. First, we established conditions to quantify by multicolor fluorescent-microscopy the content of CCL20, TNF, and VEGFA in CD68^+^ cells in FFPE melanoma tissues. To validate the staining specificity, we preincubated the anti-cytokine antibodies with an excess of each cytokine, confirming that staining was specifically blocked on FFPE melanoma tissues (Supplementary Figure S1). Figure 2A shows representative images of CD68^+^ TAMs merged with CCL20, TNF, or VEGFA of two primary melanoma samples representative of low and high cytokine expression profiles. Dot plots in Figure 2B represent quantification at the single-cell level of VEGFA and TNF MFI in accumulated TAMs from *n* = 11 non-metastasizing melanomas compared with *n* = 22 metastasizing primary melanomas, showing higher TNF/VEGFA expression by CD68^+^ TAMs in clinically aggressive melanomas. Correlations between TNF and VEGFA MFI levels (R = 0.69, *n* = 22, *p* < 0.001) or TNF and CCL20 (R = 0.62, *n* = 10, *p* < 0.001) by TAMs were found among cases of metastasizing tumors, indicating a positive relationship among these protumoral cytokines. To assess the cytokine expression profile of primary tissue cells with a different methodology, we purified TAMs and tumor cells from 4 thick metastasizing primaries (stage IV patients at diagnosis) to quantify mRNA expression, showing active gene expression of TNF, CCL20, and VEGFA by TAMs compared to peripheral blood monocytes isolated from these patients. In contrast, remaining cancer cells transcribed less *CCL20*, *TNFA*, and *VEGFA* than TAMs (Figure 2C). Next, we used the Kaplan–Meier method to assess the clinical relevance of quantifying the MFI expression level of CCL20, TNF, and VEGFA cytokines by TAMs in FFPE melanoma tissues from 83 primary melanoma patients (Figure 2D,E). Patients were stratified as ‘high’ or ‘low’, using the cell-specific median MFI value for each cytokine as the cut-off point. High TAM-CCL20 or TAM-TNF content correlated with shorter DFS and OS (log-rank test, *p* ≤ 0.01), whereas high TAM-VEGFA expression correlated only with shorter DFS (*p* = 0.005). CCL20 and TNF were not expressed by cancer cells and no correlation was found between cancer cell-VEGFA content and DFS or OS (*p* = 0.19and *p* =0.63, respectively) (Table 1 and Table S2). To determine whether TAM-specific quantification of CCL20, TNF, or VEGFA were independent prognostic factors, we performed a multivariate regression analysis including gender, age, location, histologic type, ulceration, Breslow, and stage parameters (Table 1). This analysis showed that TAM-specific CCL20 and TNF expression levels were independent prognostic factors for DFS (*p* < 0.001) and OS (*p* = 0.004 and *p* = 0.001, respectively) in this cohort. Whereas TAM-VEGFA content was an independent predictor of DFS (*p* < 0.001) but did not reach significance for OS. Finally, we analyzed the prognostic value of the CCL20/TNF/VEGFA combined secretory TAM phenotype, showing its great potential in the prediction of DFS and OS of primary melanoma patients (*p* < 0.0001 and *p* = 0.0004, respectively) (Figure 2F). Consequently, we validated our previous pilot study in a large and independent patient cohort, using standard FFPE tissue sections instead of cryosections, and confirmed the role of CCL20, TNF, and VEGFA expression by TAMs in predicting clinical behavior of primary cutaneous melanoma patients. Our results highlight the role of TAMs in biologically aggressive primary melanomas, suggesting that prometastatic TAMs are characterized by higher secretion of CCL20/TNF/VEGFA cytokines.

**Figure 3:**
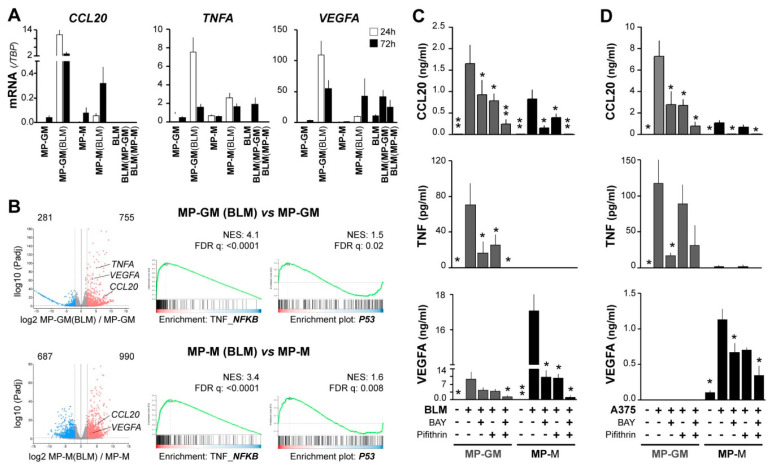
NF-κB and p53 signaling pathways synergically contribute to CCL20, TNF, and VEGFA upregulation in macrophages conditioned by melanoma cells. (**A**) Macrophages differentiated with GM-CSF or M-CSF (MP-GM and MP-M, respectively) were cocultured with BLM cells for 24 h or 72 h, as specified, and then separated with anti-CD14-coated beads. Mutually conditioned cells were measured for *CCL20*, *TNF*, and *VEGFA* mRNA expression relative to *TBP* (*n* = 3–5 donors). (**B**) Volcano plots showing DEG scores for 24h BLM-conditioned macrophages vs. unconditioned macrophages, as well as GSEA analyses showing normalized enrichment scores and false discovery rates (*n* = 3). (**C**,**D**) Conditioned media assessed for CCL20, TNF, and VEGFA protein secretion. As in A, differentiated macrophages were cocultured with BLM (**C**) or A375 (**D**) melanoma cells for 72 h in the absence or presence of 10 µM BAY11-7082 (NF-κB inhibitor) and/or 50 µM Pifithrin-α (p53 inhibitor), as indicated (*n* = 5–10). Asterisks denote statistically significant differences relative to melanoma/macrophage condition cocultured without inhibitors (paired *t*-test; * *p* < 0.05; ** *p* < 0.01).

To search for mechanisms underlying the CCL20/TNF/VEGFA secretory TAM phenotype associated with aggressive primary melanomas, and due to limited access to patient TAMs, we prepared tumor-conditioned macrophages. We previously showed that both pro-inflammatory (GM-CSF) and anti-inflammatory (M-CSF) human macrophages (MP-GM and MP-M, respectively) co-cultured with melanoma metastatic cell lines (BLM and A375), displayed a distinctive CCL20 mRNA up-regulation and secreted large amounts of CCL20 protein [17]. Similarly, we now observed up-regulation of TNF and VEGFA mRNA expression by melanoma-conditioned MP-GM(BLM) and, to a lesser amount, by MP-M(BLM), which were separated from BLM melanoma cells after co-culture (Figure 3A). Melanoma BLM cells were also analyzed after co-culture, showing no expression of CCL20 and low induction of *TNF* mRNA, whereas *VEGFA* mRNA was basally expressed by BLM cells. To get insights into the molecular programs activated by melanoma-conditioning of MP, we performed RNA sequencing on MP-GM and MP-M with and without co-culturing with BLM cells. 1036 genes were found significantly differentially expressed in melanoma-conditioned MP-GM(BLM) versus MP-GM, and 1677 genes in melanoma conditioned MP-M versus MP-M(BLM) (adjusted *p*-value < 0.05; log2 fold change >2) (Figure 3B). Pathway analysis identified a number of pathways related to TNF_NF-κB and p53 signaling in the melanoma-conditioned MPs (Figure 3B).

To explore the effect of targeting these pathways during co-cultures of MPs (GM and M) with melanoma cells, we used BLM and A375 (which do not express VEGFA basally) metastatic melanoma cell lines, which similarly induced secretion of the three cytokines in the co-cultures (Figure 3C,D). However, there were differences in the cytokine profile up-regulated regarding the MP polarization profile; MP-GM/melanoma produced more TNF and CCL20, whereas MP-M/melanoma produced more VEGFA. Next, we used BAY 11-7082 to inhibit the NF-κB signaling cascade and the p53 inhibitor pifithrin and analyzed secretion of CCL20, TNF, and VEGFA proteins in the supernatants; getting additive inhibitory effects in the secretion of the three cytokines. This data indicate that macrophage-melanoma interactions induced both signaling pathways, NF-κB and p53 activation, which may contribute to up-regulate the specific cytokine secretory phenotype of TAMs, regarding their previous M1/M2 polarization heterogeneity.

## 4. Discussion

Because TAMs are a very heterogeneous population of immune cells, which has been reported to be associated with either anti-tumoral or protumoral functions, assessment of their molecular or functional variety may provide new biomarkers for patient prognosis or to predict treatment responses [6,10]. Accurate prognosis in melanoma is particularly important to identify those patients who might benefit from adjuvant treatment. In the present study, we have quantitatively evaluated conventional parameters like density, morphology, stroma/tumor nest distribution, as well as more in the deep characterization of TAM polarization and cytokine content. In clinically relevant primary melanoma patient cohorts, we did not find a significant association of TAM density, size, and location, nor CD11c/CD209 subsets with DFS or OS. In prior work, our group identified a particular secretory phenotype of TAMs isolated from melanoma tissues [17]. Melanoma TAMs were also characterized in situ by their high content of CCL20, TNF, and VEGFA by multicolor immunofluorescence in cryopreserved samples; we now show that this particular cytokine profile may be detected and quantified in diagnostic FFPE. Importantly, the high content of CCL20/TNF/VEGFA is strongly associated with a worse prognosis in primary melanoma patients.

Nowadays it is well recognized that the interaction of the cancer cells with other cells or factors in the TME has an impact on tumor biology [16]. A deep analysis of TME may provide clues to understanding these complex relationships and, ultimately, will be of clinical utility for the establishment of novel prognostic tools or therapeutic targets. To this end, we previously used multicolor immunofluorescence microscopy to study in situ diverse immune and stromal components, as well as the expression of chemokines and their receptors in the TME of human melanoma. This non-destructive tissue methodology allowed us to study the spatial distribution of diverse cells, location of specific proteins within cells or tissue areas, and quantification of their relative level of expression. Human tissue studies commonly use a chromogenic IHC approach to evaluate specific proteins or cell markers in situ; however, only single or double-labeling are suitable. Fluorescent IHC and spectral microscopy have the advantage of multiple marker detection; accordingly, it was used to analyze immune infiltrates in primary melanomas, showing that combination assessment of CD8^+^ lymphocytes/CD68^+^ macrophages ratio in the stroma correlated with shortened survival [28]. We used a similar approach, combined with confocal microscopy and image processing, to quantify at the cellular level diverse TAM characteristics in melanoma. Multiple labeling is of particular relevance when evaluating heterogeneous cells like macrophages, which are known to share markers with other myeloid cells or to express molecules in relation to their functional polarization. As an example, CD11c, a marker traditionally associated with dendritic cells (DCs), is also expressed by M1 macrophages [29]. In previous studies, we established that TAMs are the most abundant type of myeloid cells in melanoma tissues, as compared with other mononuclear (CCR2^+^ monocytes or CD1c^+^ myeloid DCs) and polymorphonuclear phagocytes [17,19]. In this study, we set up conditions for multilabeling and quantitative image analysis of TAM markers in both cryopreserved and paraffin-embedded tissues, which are more convenient for clinical utility. First, as bona fide pan-macrophage markers, we showed that CD163, CD68, and CD115 were co-expressed by most cells with macrophage morphology. CD163 did not define any separated subset of TAMs, which is in agreement with its co-expression with both M1 and M2 markers in Th1 and Th2-predominant pathologies [11]. Definition of TAM subpopulations was better achieved by triple staining with a pan-macrophage marker to gate macrophages, and quantitative assessment of the level of expression of CD11c and CD209. Thus, dot-plot analysis of CD11c versus CD209 expression at the single-cell level showed that in control skin most macrophages were CD209^+^CD11c^−^ cells, which may represent tissue-resident macrophages [30]. By contrast, melanoma tissues contained a novel CD11c^+^CD209^−^ subset, maybe representing incoming proinflammatory macrophages, and CD11c^+^CD209^+^ mixed cells. However, no major differences were observed between clinically divergent groups regarding CD11c/CD209 subsets or other TAM parameters as density, morphology, or location.

Cytokine production by macrophages is a hallmark of their polarized function [9]; M1 release large amounts of proinflammatory cytokines (such as IL-12, IL-23, and TNF), whereas M2 are characterized by high expression of IL-10 and proangiogenic growth factors such as IL-8, VEGFA, and VEGFC. Interestingly, CCL20 secreting TAMs were induced via IL-6-regulated macrophage polarization in a mouse model of colon cancer; in which, CCL20 promoted cancer progression by recruiting CCR6^+^ lymphocytes [31]. Macrophage derived-TNF was identified as a crucial melanoma growth factor that contributed to resistance to MAPK pathway inhibitory treatments in a mouse model of Braf^V600E^ mutated melanoma [32]. We had previously identified the expression of CCL20/TNF/VEGFA cytokines by TAMs in a pilot study with cryopreserved human melanoma tissues [17]. Because FFPE tissues are the current method for patient sample preservation, we validated our multiple-labeling methodology to analyze TAM cytokine signature in 83 FFPE samples from a second patient cohort. Importantly, CCL20/TNF/VEGFA cytokine production by TAMs correlated with poor patient survival. Using monocyte-derived macrophages, with either GM-CSF or with M-CSF, which are known to prime towards M1 or M2 phenotypes, we produced in vitro melanoma-conditioned macrophages expressing CCL20 and TNF (M1-primed) or VEGFA (M2-primed). This in vitro culture system of macrophages with melanoma cells, allowed us to explore the pathways underlying the CCL20/TNF/VEGFA cytokine secretory phenotype of TAMs, showing that p53 and NF-κB coregulate tumor-conditioned macrophages. It is known that NF-κB activation drives M2 polarization of TAMs [33] and its inhibition re-educates TAMs towards an anti-tumoral phenotype [34]. In accordance with our results, dual p53 and NF-κB activation in macrophages were described to up-regulate IL6, TNF, and CCL20 [35]. These results identify a unique p53/NF-kB pathway in protumoral TAMs that could be targetable to re-educate TAMs to block their protumoral functions.

## 5. Conclusions

Our studies show that CCL20/TNF/VEGFA cytokine production by TAMs is a hallmark of their protumoral deviation. Assessment of TAM cytokine profile in situ by multicolor staining of FFPE tissues is feasible and may be used as a new prognostic marker in cutaneous melanoma. In addition, we identify p53 and NF-κB as the main pathways driving the skewed production of CCL20/TNF/VEGFA cytokines by TAMs, pointing to the development of new therapeutic targets to re-educate this particular deviation of TAMs and to promote their anti-tumoral function.

## Figures and Tables

**Table 1:** Univariate and multivariate Cox regression analyses for 10-year disease-free and overall survival (FFPE cohort).

Univariate	Disease-Free Survival			Overall Survival		
Gender (F vs. M)	1.602	0.91–2.81	0.100	1.168	0.55–2.47	0.685
Age (years)	1.003	0.99–1.02	0.704	1.007	0.98–1.03	0.574
Location (H/L vs. T)	1.452	0.74–2.85	0.278	0.895	0.40–1.99	0.785
Subtype (nod vs. others)	0.831	0.46–1.52	0.547	1.305	0.60–2.82	0.498
Ulceration (yes vs. no)	1.354	0.76–2.41	0.301	2.582	1.15–5.82	0.022
Breslow (mm)	1.130	1.07–1.20	<0.001	1.124	1.04–1.21	0.002
Stage (II vs. III–IV)	1.865	1.19–2.92	0.006	1.793	0.74–4.33	0.194
TAM-CCL20 (MFI, each 10 a.u.)	1.195	1.10–1.29	<0.001	1.164	1.05–1.29	0.003
TAM-TNF (MFI, each 10 a.u.)	1.295	1.18–1.42	<0.001	1.250	1.11–1.41	0.000
TAM-VEGFA (MFI, each 10 a.u.)	1.185	1.11–1.26	<0.001	1.051	0.96–1.15	0.264
TC-VEGFA (MFI, each 10 a.u.)	1.050	0.98–1.13	0.189	1.026	0.92–1.14	0.629
**Multivariate**	**HR**	**95% CI**	***p***	**HR**	**95% CI**	***p***
Gender (F vs. M)	1.019	0.56–1.87	0.951	0.612	0.25–1.47	0.274
Age (years)	0.999	0.98–1.02	0.861	1.010	0.99–1.03	0.387
Breslow (mm)	1.164	1.09–1.25	<0.001	1.173	1.06–1.30	0.002
Stage (II vs. III–IV)	0.716	0.33–1.53	0.390	0.596	0.17–2.10	0.421
TAM-CCL20 (MFI, each 10 a.u.)	1.244	1.12–1.38	<0.001	1.260	1.08–1.47	0.004
Gender (F vs. M)	1.245	0.71–2.20	0.449	0.666	0.27–1.64	0.376
Age (years)	0.996	0.98–1.01	0.692	1.005	0.98–1.03	0.715
Breslow (mm)	1.115	1.05–1.18	<0.001	1.123	1.03–1.22	0.008
Stage (II vs. III–IV)	1.401	0.90–2.19	0.138	1.013	0.35–2.92	0.982
TAM-TNF (MFI, each 10 a.u.)	1.304	1.19–1.43	<0.001	1.255	1.09–1.44	0.001
Gender (F vs. M)	1.103	0.61–2.00	0.747	0.811	0.34–1.91	0.632
Age (years)	1.005	0.99–1.02	0.577	1.008	0.98–1.03	0.550
Breslow (mm)	1.138	1.06–1.22	<0.001	1.124	1.03–1.23	0.008
Stage (II vs. III–IV)	1.122	0.63–1.99	0.694	1.230	0.46–3.32	0.683
TAM-VEGFA (MFI, each 10 a.u.)	1.188	1.11–1.27	<0.001	1.040	0.95–1.14	0.404

## Data Availability

Available at https://www.ncbi.nlm.nih.gov/geo/query/acc.cgi?acc=GSE171277 (accessed on 4 August 2021).

## Supplementary Materials

The following are available online at https://www.mdpi.com/article/10.3390/cancers13163943/s1, Figure S1: Validation of staining specificity, Table S1: Frozen and paraffin-embedded primary melanoma collections, clinicopathological features, Table S2: Association of evaluated prognostic markers with clinicopathological features (Mann-Whitney).
