# Supplementary figures and images for: CCL20/TNF/VEGFA Cytokine Secretory Phenotype of Tumor-Associated Macrophages Is a Negative Prognostic Factor in Cutaneous Melanoma
Source: Cancers (Basel). 2021 Aug 5;13(16):3943. doi: 10.3390/cancers13163943 (PMC8392234; doi:10.3390/cancers13163943)

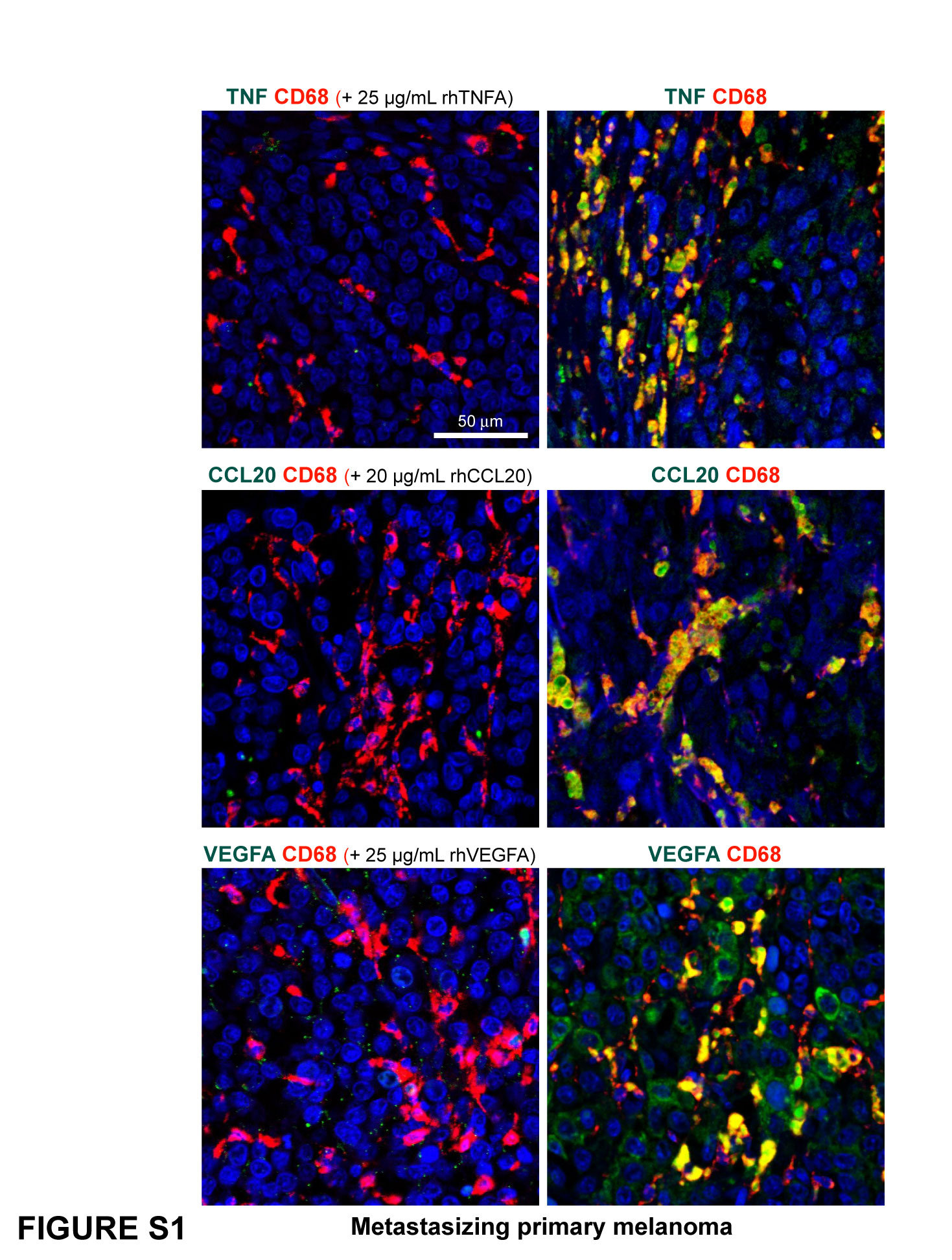

Supplement: Supplementary file 1 [file cancers-13-03943-s001.zip › FIG S1 rev 8-21.jpg]
